# Infection tuberculeuse latente chez l’enfant à Bangui: à propos de 524 cas exposés à domicile aux cas index de tuberculose pulmonaire à microscopie positive

**DOI:** 10.11604/pamj.2021.40.263.32064

**Published:** 2021-12-27

**Authors:** Edgard Djimbélé Béradjé, Alain Farra, Jess Elio Kosh Komba Palet, Marie Colette Nganda- Bangué, Simplice Cyriaque Kango, Boniface Koffi, Jean Chrysostome Gody

**Affiliations:** 1 Service de Pédiatrie, CHU Domitien de Bimbo, Bangui, République Centrafricaine,; 2 Laboratoire National de Référence de la Tuberculose, Institut Pasteur de Bangui, Bangui, République Centrafricaine,; 3 Centre Hospitalier Universitaire Pédiatrique de Bangui, Bangui, République Centrafricaine,; 4 Service d´Anatomie Pathologie, Laboratoire National de Biologie Clinique et de Santé Publique de Bangui, Bangui, République Centrafricaine

**Keywords:** Infection tuberculeuse latente, contacts, cas index, domicile, Bangui, Latent TB infection, contacts, index cases, household, Bangui

## Abstract

**Introduction:**

malgré la vaccination par le BCG, le risque de développer la tuberculose chez les jeunes enfants dans les régions endémiques reste plus élevé après exposition au cas de tuberculose chez l´adulte. Le but de cette étude était de réduire le risque de tuberculose active chez les enfants contacts des cas index adultes à domicile.

**Méthodes:**

une étude transversale multisite (avril 2016-janvier 2019) a été réalisée sur des enfants de 0 à 59 mois, contacts des cas index à domicile, dépistés et suivis au centre pédiatrique de Bangui.

**Résultats:**

cinq cent vingt- quatre (524) enfants ont été enregistrés. La moyenne d´âge était de 2 ans et 1 mois et le sex ratio garçon/fille de 1,02. Environ quatre-vingt-huit pourcent (88,5%) des contacts étaient vaccinés avec le BCG contre 11,5% non vaccinés. Dans plus de la moitié des cas (52%), les contacts et les cas index ont partagé la même chambre et le temps de contact journalier était supérieur à 12h dans 56% des ménages. Un peu plus de neuf pourcent (9,35%) des contacts avaient une IDR positive. Tous les enfants ont été mis sous chimioprophylaxie à base de Rifampicine + Isoniazide selon les directives nationales et malgré cela, 14 soit 2,67% ont développé une tuberculose active dont 13 formes pulmonaires et une forme ganglionnaire.

**Conclusion:**

la chimio prophylaxie tuberculeuse a sans doute permis de réduire considérablement le risque de survenue de la tuberculose chez des enfants ayant été en contact avec les cas index à domicile.

## Abstract (en)

**Introduction:**

in endemic areas, despite BCG vaccination, the risk of developing tuberculosis (TB) in young children is high after exposure to adults with tuberculosis. The purpose of this study is to reduce the risk of active tuberculosis in children experiencing household exposure to adult index cases.

**Methods:**

we conducted a cross-sectional multi-site study (April 2016- January 2019) of children aged 0 to 59 months experiencing household exposure to index cases. They were screened and followed up at the pediatric center in Bangui.

**Results:**

five hundred twenty four children were included in the study. The average age of patients was 2 years and 1 month and sex ratio (male/female) was 1.02; more than eighty-eight percent (88.5%) of contacts had received a BCG vaccination versus 11.5% who were unvaccinated. In more than half of the cases (52%), contacts and index cases had shared the same room and daily contact time had been greater than 12h in 56% of households; more than nine percent (9.35%) of contacts had positive tuberculin skin (IDR) test. All children received chemoprophylaxis with rifampicin + isoniazid, according to the national guidelines and, despite this, 14 or 2.67% of patients developed active tuberculosis, including 13 patients with pulmonary tuberculosis and one with ganglionic tuberculosis.

**Conclusion:**

chemoprophylaxis of tuberculosis significantly reduced the risk of TB in children experiencing household exposure to index cases.

## Introduction

La tuberculose est l´une des dix importantes causes de décès et la principale cause due à un seul agent infectieux avant le VIH/SIDA. En 2017, elle a causé 1,3 millions de décès parmi les personnes séronégatives pour le VIH et 300 000 décès supplémentaires parmi les personnes séropositives pour le VIH. L´incidence est estimée à 10 millions de cas dont 1 million d´enfants [1]. Selon l´OMS, environ 1,7 milliard soit 23% des populations du monde sont susceptibles d´avoir une infection tuberculose latente et donc à risque de développer une tuberculose active durant leur vie. L´enfant représente du fait de sa relative fragilité un terrain de prédilection pour la primo-infection tuberculeuse [2]. Dans les régions endémiques, malgré la vaccination par le BCG, le risque de développer la tuberculose chez les jeunes enfants est plus élevé après exposition au cas de tuberculose chez l´adulte [3,4]. Le développement de la tuberculose chez les enfants a tendance à se faire peu après l´infection. Beaucoup de cas surviennent dans les 6 mois après l´infection et la grande majorité dans les 12 mois [5]. Aussi, le risque de la tuberculose chez les enfants est plus élevé dans les pays de niveau socio- économique bas, dans les cas de la malnutrition, de l´infection à VIH et dans les cas où la prévalence de la tuberculose est élevée chez l´adulte. Diverses études ont démontré que la tuberculose chez l´enfant pourrait être prévenue si l´on avait mieux appliqué les activités de base de lutte contre la tuberculose [5].

La RCA reste l´un des pays ayant une forte charge tuberculeuse avec une incidence de 540 pour 100 000 habitants et une mortalité de 61 pour 100 000 habitants [6]. Selon les dernières études, la tuberculose pharmaco résistante représente 4,1% parmi les nouveaux cas et 25,2% parmi les cas déjà traités [7]. Les lignes directrices actuelles de l´OMS sur l´infection tuberculeuse latente en 2018 sont fondées sur la progression vers la tuberculose maladie évolutive dans les groupes à risques spécifiques. Ainsi, selon les directives du Programme Nationale de Lutte contre la Tuberculose, les enfants de moins de 5 ans ayant été en contact avec un cas de tuberculose pulmonaire bactériologiquement confirmé et qui ne présentent aucun signe de tuberculose active doivent recevoir un traitement préventif à la rifampicine et l´isoniazide pendant 3 mois [8]. Au regard de ce qui précède, la présente étude avait pour but de réduire le risque de développement de la tuberculose active chez les enfants ayant été en contact avec les cas index adultes de tuberculose pulmonaire à microscopie positive (TPM+) à Bangui.

## Méthodes

**Type, durée et lieu de l´étude:** il s´agit une étude transversale descriptive d´une durée de 2 ans et 9 mois (avril 2016-janvier 2019). Le cadre de l´étude était constitué de 4 centres de diagnostic et de traitement (CDT) de la tuberculose où les cas ont été recrutés et le CHU Pédiatrique de Bangui (CHUPB) pour leur suivi. Les CDT étaient ceux de l´Hôpital de l´Amitié, du Camp Fidèle Obrou, de Boy-Rabe et de Bede-Combattant.

**Population d´étude:** elle était constituée des enfants des deux sexes âgés de 0 à 59 mois ayant été en contact à domicile des cas index dépistés dans les quatre CDT et dont les suivis ont été faits au CHUPB. L´échantillonnage a été exhaustif prenant en compte tous les contacts enregistrés pendant l´étude.

**Critères d´inclusion:** tous les enfants de 0 à 59 mois ayant été en contact à domicile avec un cas index depuis au moins trois mois, ne présentant pas les signes cliniques d´une tuberculose active pour lesquels nous avons obtenu le consentement des parents.

**Critères de non inclusion:** les enfants ayant été en contact à domicile avec le cas index et présentant les signes cliniques d´une tuberculose active, le non consentement des parents et les perdus de vue.

**Collecte des données:** les données cliniques et sociodémographiques des patients ont été collectées à partir de 3 fiches différentes selon les cas de figure: une fiche d´enquête pour les cas contact index au niveau des CDT, une 2^e^ fiche ayant servi pour les visites à domicile concernant les sujets contacts et enfin, une 3^e^ fiche pour le suivi des enfants au niveau du CHUPB.

**Analyse des données:** les données ont été analysées avec le logiciel EPI- Data version 2017 et le tableur Excel a permis de réaliser les figures et les graphiques.

**Aspects éthiques:** ce protocole d´étude a été approuvé par un comité d´experts du ministère de la santé pour être réalisé en routine dans les formations sanitaires pour le compte du Programme national de lutte contre la tuberculose. Les consentements des cas index ont été obtenus et ceux des enfants via leurs parents ou leurs tuteurs. Les données ont été exploitées dans la stricte confidentialité et l´anonymat préservant l´identité des patients.

## Résultats

**Caractéristiques sociodémographiques:** au cours de cette étude, 524 enfants ayant été en contact avec des cas index présentant une TPM+ ont été enregistrés. La moyenne d´âge était de 2 ans et 1 mois avec des extrêmes allant de 10 jours à 4 ans et 9 mois. Soixante deux pourcent (62%) avaient moins de 3 ans et 38% étaient âgés de plus de 3 ans. Il y avait presque autant de garçon (50,6%) que de fille (49,4%) avec un sex ratio garçon fille de 1,02. Tous les contacts ont partagé une maison commune avec le cas index et 85% de ces sujets étaient vaccinés avec le BCG contre 11,5% non vaccinés (Tableau 1).

**Tableau 1:** aspects sociodémographiques des enfants contacts des cas index à domicile enregistrés d´avril 2016 à janvier 2019 (N = 524)

Statut sociodémographique	Nombre	Pourcentage (%)
**Age (an)**		
≤ 3	323	62
≥ 3	201	38
**Sexe**		
M	265	50,6
F	259	49,4
**Vaccin BCG**		
Oui	464	88.5
Non	60	11.5

*BCG: Bacille de Calmette et Guérin

**Nombre de contact et types d´activités du contact avec le cas index:** plus de 84% des contacts avec le cas index ont eu lieu tous les jours, le plus souvent au cours des repas (69,66%) et dans 52%, le sujet contact et le cas index ont partagé la même chambre (Tableau 2).

**Tableau 2:** répartition des circonstances et des nombres de contact entre les enfants contacts et les cas index enregistrés pendant la période d´avril 2016 à janvier 2019 (N = 524)

Nombre de contact	Circonstances de contact		
	**Jeux**	**Repas**	**Chambre**
**Une fois/semaine**	3(0,57%)	51(9,73%)	221(42,18%)
**Deux fois/semaine**	8(1,53%)	41(7,82%)	23(4,39%)
**Plusieurs fois/semaine**	69(13,17%)	67(12,79%)	8(1,53%)
**Tous les jours**	444(84,73%)	365(69,66%)	272(51,91%)
**Total**	524 (100,0%)	524 (100,0%)	524 (100,0%)

**Durée de contact avec le cas index:** dans plus de la moitié des cas (56%), le temps de contact journalier avec le cas index était supérieur à 12h (Tableau 3).

**Tableau 3:** nombre d´heures d´exposition journalière entre les cas index et les enfants contacts à domicile enregistrés pendant la période d´avril 2016 à janvier 2019

Nombre d´heures d´exposition journalière	Effectif (%)
< 4 h	13(2,48)
4- 12 h	215 (41,03)
>12 h	296(56,49)
Total	524(100)

**Résultats de l´IDR réalisés chez les contacts:** plus de 90% des contacts avaient une IDR négative. Un peu plus de neuf pourcent (9,35%) des contacts avaient une IDR positive dont 5,73% avec une réponse phlycténulaire (> 15 mm) (Tableau 4).

**Tableau 4:** résultats des tests IDR effectués chez les enfants contacts des cas index à domicile enregistrés pendant la période d´avril 2016 à janvier 2019

< 10 mm	475(90,64)	
10-15	19(3,63)	9,35%
> 15 mm	30(5,73)	
Total	524(100)	

*IDR: Intra Dermo Réaction

**Evolution des enfants sous la chimioprophylaxie:** tous les enfants ont été mis sous chimioprophylaxie à base de Rifampicine + Isoniazide selon les directives nationales. 514 soit 98,1% l´ont correctement suivi alors que 10 d´entre eux soit 1,9% l´ont abandonné avant le terme c´est-à-dire la fin du 3ème mois. Au cours du suivi, 99 enfants ont été référés à la consultation du médecin pour suspicion de la tuberculose évolutive. L´examen clinique avait montré une altération de l´état général dans 37,4% (37/99), des adénopathies superficielles dans 4,04% (4/99) et les difficultés respiratoires dans 3,03% (3/99). Sur la base des arguments cliniques, radiologiques et devant l´échec d´une antibiothérapie non spécifique en plus de la chimioprophylaxie, 14 sur les 524 soit 2,67% ont été reconnus comme ayant une tuberculose active dont 13 formes pulmonaires et une forme ganglionnaire.

## Discussion

L´intérêt de cette étude était d´une part de contribuer à la prévention du développement de la tuberculose chez des enfants de moins de cinq ans ayant été en contact avec des adultes souffrant de la TPM+ et d´autre part de comparer l´évolution des cas contacts aux résultats d´études similaires réalisées dans d´autres pays. Le CHUPB où nous avons réalisé cette étude est le seul hôpital de référence du pays et en particulier pour la capitale Bangui où les enfants sont hospitalisés. Les résultats obtenus dans cette étude peuvent donc refléter la situation réelle des enfants ayant été exposés à des cas de TPM+ dans la ville de Bangui.

**Age des patients:** l´étude a montré que la tranche d´âge la plus représentée était celle de moins de 3 ans (61,64%). Ces résultats sont similaires à ceux rapportés en France qui avaient montré que l´un des principaux facteurs de risque de la tuberculose est le jeune âge de l´enfant et que d´autres résultats ont soutenu le fait que ce risque est encore plus majoré chez les moins de 2 ans après exposition ce qui justifie la prophylaxie systématique dans cette tranche d´âge [9,10].

**Lien des enfants avec le cas index:** la majorité des enfants (51,53%) avait un lien de premier degré avec le cas index, ce qui a expliqué tout le risque d´exposition de ces enfants contacts. Effectivement, il a été rapporté que le lien de premier degré avec le contaminateur augmente significativement le risque de développer la maladie tuberculeuse [11,12]. Cela peut s´expliquer par le fait que le jeune âge des enfants permet un contact permanent et direct avec les parents contaminateurs et les expose au risque de contamination.

**Le type et la durée du contact avec le cas index:** tous les enfants inclus dans cette étude avaient une maison commune avec le cas index. Les types de contacts rapportés ici étaient le partage des jeux suivi des repas et le partage des chambres avec en particulier dans 56% plus de temps (> 12 heures) de contact avec le cas index (tableau II et III). Il a été rapporté que la proximité géographique de l´enfant avec le malade dans le ménage est un risque réel de même lorsque le temps passé avec le cas index est prolongé et plus encore lorsque le contact est nocturne en cas de partage de chambre [12,13].

**Vaccination BCG:** environ quatre-vingt-huit pour cent (88,5%) de la population d´étude était vaccinée contre la tuberculose et présente les cicatrices de la vaccination. Quand bien même le BCG est gratuit dans nos formations sanitaires et administré à tout nouveau-né avant la sortie de la maternité sauf contre-indication, 11,5% des enfants n´avaient pas de preuve de vaccination. Certaines études ont rapporté que le risque d'attraper la tuberculose est quatre fois plus élevé chez l'enfant non vacciné mais que malgré le statut vaccinal, les cas de tuberculose chez les enfants vaccinés ne sont pas nuls [2].

**Diagnostic:** le diagnostic de la tuberculose chez l´enfant a nécessité en plus de l´exposition à un cas de TPM+, la recherche des signes évocateurs, les arguments radiologiques et les résultats de l´IDR et ceux de laboratoires [14]. La forme pulmonaire reste classiquement la plus représentée [15]. Malgré la chimioprophylaxie, 2,7% des enfants ont développé la maladie. En absence de la chimio prophylaxie, 10% des sujets atteints d´ITL pourraient développer la TB active [10]. Cette étude vient donc conforter l´apport de la chimio prophylaxie dans la réduction des risques de progression vers la tuberculose maladie.

## Conclusion

La mise sous chimio prophylaxie tuberculeuse a permis de réduire le risque de la progression vers la maladie chez des enfants ayant été au contact à domicile des cas de tuberculose pulmonaire à microscopie positive. En vue d´améliorer la prise en charge des enfants contacts, une sensibilisation de la population sur le risque d´évolution de cette maladie est importante de même que le recyclage du personnel soignant et la vulgarisation des directives nationales sur la chimio prophylaxie tuberculeuse.

### Etat des connaissances sur le sujet

- *Les cas de tuberculose chez l´enfant de moins de 5 ans après exposition au cas index adulte à domicile ont déjà été documentés*.

### Contribution de notre étude à la connaissance

- *La chimioprophylaxie tuberculeuse chez les enfants de moins de 5 ans n´était pas encore pratiquée en RCA bien que les directives nationales existent en cette matière;*
- *Ce travail avait donc servi d´études pilote et à l´issue de cette étude, les enfants de moins de 5 ans contacts des cas index adultes à domicile sont désormais investigués, suivis, et mis sous chimioprophylaxie antituberculeuse*.

## Remerciements

Nous remercions le Dr Koura Kobto Ghislain de l´Union Internationale contre la Tuberculose qui a été superviseur du projet d´étude TITI en RCA dont la mise en place a permis de réaliser cette étude. Nous remercions le personnel des CDT ainsi que tous les enfants dont leur inclusion a permis la réalisation de cette étude.
